# Optical coherence tomography angiography in diabetic retinopathy

**DOI:** 10.1016/j.preteyeres.2023.101206

**Published:** 2023-07-26

**Authors:** Nadia K. Waheed, Richard B. Rosen, Yali Jia, Marion R. Munk, David Huang, Amani Fawzi, Victor Chong, Quan Dong Nguyen, Yasir Sepah, Elizabeth Pearce

**Affiliations:** a New England Eye Center, Tufts University School of Medicine, Boston, MA, USA; b New York Eye and Ear Infirmary of Mount Sinai, Icahn School of Medicine at Mount Sinai, New York, NY, USA; c School of Medicine, Casey Eye Institute, Oregon Health and Science University, Portland, OR, USA; d Augenarzt-Praxisgemeinschaft Gutblick AG, Pfȧffikon, Switzerland; e Department of Ophthalmology, Feinberg School of Medicine, Northwestern University, Chicago, IL, USA; f Institute of Ophthalmology, University College London, London, UK; g Byers Eye Institute, Stanford University School of Medicine, Stanford, CA, USA; h Boehringer Ingelheim Pharmaceuticals Inc., Ridgefield, CT, USA

**Keywords:** Diabetic macular edema, Diabetic macular ischemia, Diabetic retinopathy, Optical coherence tomography, Optical coherence tomography angiography

## Abstract

There remain many unanswered questions on how to assess and treat the pathology and complications that arise from diabetic retinopathy (DR). Optical coherence tomography angiography (OCTA) is a novel and non-invasive three-dimensional imaging method that can visualize capillaries in all retinal layers. Numerous studies have confirmed that OCTA can identify early evidence of microvascular changes and provide quantitative assessment of the extent of diseases such as DR and its complications.

A number of informative OCTA metrics could be used to assess DR in clinical trials, including measurements of the foveal avascular zone (FAZ; area, acircularity, 3D para-FAZ vessel density), vessel density, extrafoveal avascular zones, and neovascularization. Assessing patients with DR using a full-retinal slab OCTA image can limit segmentation errors and confounding factors such as those related to center-involved diabetic macular edema. Given emerging data suggesting the importance of the peripheral retinal vasculature in assessing and predicting DR progression, wide-field OCTA imaging should also be used. Finally, the use of automated methods and algorithms for OCTA image analysis, such as those that can distinguish between areas of true and false signals, reconstruct images, and produce quantitative metrics, such as FAZ area, will greatly improve the efficiency and standardization of results between studies.

Most importantly, clinical trial protocols should account for the relatively high frequency of poor-quality data related to sub-optimal imaging conditions in DR and should incorporate time for assessing OCTA image quality and re-imaging patients where necessary.

## 1. Introduction

Diabetic retinopathy (DR) is the most common complication of diabetes mellitus and a leading cause of vision loss in people of working age, with an estimated global prevalence of 27–35% among all patients with diabetes (Lee et al., 2015; Simó-Servat et al., 2019; Thomas et al., 2019). DR has a high estimated global burden: in 2021, it was projected that patients with DR would exceed 160 million by 2045 (Teo et al., 2021).

Although there is a long history of research into DR (Wolfensberger and Hamilton, 2001), there remain many unanswered questions about how to assess and treat the pathology and complications that arise from this disease (Wong and Sabanayagam, 2020), including how DR may differentially affect different layers of the retina, how current (and future) treatments affect the retinal microvasculature and therefore impact retinal blood flow, and how functional measures link to anatomical and vascular metrics. Optical coherence tomography angiography (OCTA) is an emergent imaging modality that may be able to provide information that answers some of these questions (de Barros Garcia et al., 2017).

## 2. Diabetic retinopathy and its complications

The early clinical features of DR occur because of chronic hyperglycemia leading to changes in the vascular wall such as pericyte loss, as well as microvascular damage, which includes occlusion of the capillaries as well as increased leakage from these vessels into surrounding tissue and subsequent abnormalities in blood flow. These events lead to progressive microvascular injury, resulting in microaneurysms, hard exudates, and retinal hemorrhages (Lechner et al., 2017). As DR progresses, a range of complications may manifest, including cotton wool spots, venous beading, and intraretinal microvascular abnormalities (IRMAs) (Lechner et al., 2017). Furthermore, with the onset of proliferative DR, retinal neovascularization can emerge, leading to serious complications such as preretinal and vitreous hemorrhages, as well as tractional retinal detachment (Lechner et al., 2017). These complications can cause severe vision loss and may necessitate vitrectomy surgery. Patients may develop additional complications that exacerbate vision loss, including diabetic macular edema (DME) and diabetic macular ischemia (DMI) (Miller et al., 2017; Sim et al., 2014). Advanced, proliferative DR is typically treated with panretinal photocoagulation, alongside anti-vascular endothelial growth factor (VEGF) therapies, depending on specific comorbidities (Gonzalez et al., 2021; Solomon et al., 2017).

DME is the result of a build-up of intracellular and/or extracellular fluid (edema) in the macula, originating from leaky blood vessels and the breakdown of the blood-retinal barrier. If left unmanaged, DME can result in up to 8% of vision being lost per year (Miller and Fortun, 2018) over a 3-year period; this is equivalent to 15 Early Treatment Diabetic Retinopathy Study (ETDRS) letters (Browning et al., 2018). The standard of care for DME has historically been focal laser photocoagulation. However, anti-VEGF therapies are now the gold standard for center-involved DME (Urbano, 2019), leading to a more rapid resolution of DME and results in a mean visual gain that is superior to laser treatment.

Macular ischemia is a key feature of DR (Hwang et al., 2016a) and another cause of vision loss associated with DR. It is associated with decreased macular retinal vessel density and/or non-perfusion of the superficial vascular complex (SVC) and deep vascular complex (DVC) (Abdelshafy and Abdelshafy, 2020; Bradley et al., 2016; de Barros Garcia et al., 2017; Gildea, 2019; Scarinci et al., 2019). It often results in enlargement and disruption of the foveal avascular zone (FAZ), the integrity of which is central to maintaining normal visual acuity (Balaratnasingam et al., 2016; Provis et al., 2013; Sim et al., 2013a). Irreversible vision loss in DMI occurs due to damage to neuronal tissues from macular capillary loss or capillary non-perfusion (Conrath et al., 2005; Liew et al., 2015; Sim et al., 2013a; Sorour et al., 2018). Currently, there is no formal clinical definition for DMI and no approved treatment. Treatments used to treat DME do not appear to improve ischemia (Cheung et al., 2022; Kiire et al., 2015; Munk et al., 2022).

Retinal non-perfusion in eyes with DR occurs mostly in the mid and far periphery, with up to 70% of non-perfusion occurring outside the posterior pole of diabetic eyes (Niki et al., 1984; Shimizu et al., 1981; Silva et al., 2022). Analysis of Protocol AA data showed that increasing baseline retinal non-perfusion index on ultra-widefield fluorescein angiography (UWF-FA) is associated with worsening of Diabetic Retinopathy Severity Scale (DRSS) score, development of proliferative diabetic retinopathy (PDR), and vitreous hemorrhage. These associations are driven by non-perfusion located primarily in the posterior pole (Silva et al., 2022). Ofuji et al. showed that a higher baseline retinal non-perfusion index (NPI) on UWF-FA is associated with later worsening of DRSS score, development of PDR, and vitreous hemorrhage. This is supported by data from a large, prospective longitudinal study, which confirmed that greater baseline peripheral non-perfusion and the presence of predominantly peripheral lesions (PPLs; which refer to visible abnormalities such as microaneurysms and hemorrhages) on UWF-FA are associated with a significantly higher risk of disease worsening; this included areas of both retinal non-perfusion and neovascularization. However, no association between PPLs was found in color fundus photos and an increased risk of disease worsening could be detected (Marcus et al., 2022). Ofuji et al. also found that the NPI in the posterior pole was most strongly associated with progression to PDR (Ofuji et al., 2022). A greater extent of peripheral non-perfusion is associated with greater DR severity and worse visual acuity (Antaki et al., 2020). Furthermore, a large peripheral area of capillary non-perfusion at baseline is predictive of non-response to panretinal photocoagulation, the standard treatment for proliferative DR (Torp et al., 2019). Whereas central ischemia tends to result in DMI, peripheral ischemia can lead to reactive neovascularization and further ischemia in a vicious cycle. Furthermore, neovascularization of the optic disc is linked to larger overall areas of retinal non-perfusion (Leal et al., 2007; Nicholson et al., 2019). Thus, depending on the location and severity of microvascular damage to the retina, a range of different comorbidities may arise in eyes with DR.

## 3. Optical coherence tomography angiography

OCTA is a novel and non-invasive three-dimensional (3D) imaging method that can visualize capillaries in all retinal layers (de Barros Garcia et al., 2017; Gao et al., 2016b). Detailed descriptions of the principles underpinning OCTA are beyond the scope of the current review but have been thoroughly described in other papers (de Carlo et al., 2015; Jia et al., 2015; Kashani et al., 2017; Spaide et al., 2018). In short, an OCTA image is a map constructed from blood cell motion across the retina revealed by changes in optical coherence tomography (OCT) signal intensity (de Carlo et al., 2015; Jia et al., 2015).

Numerous studies have confirmed that OCTA can be used to identify the earliest evidence of microvascular change and provide quantitative assessment of the extent of diseases such as DR and its common complications, including DMI (de Carlo et al., 2016; Hwang et al., 2016a, 2018; Rosen et al., 2019; Salz et al., 2016). OCTA has been shown to be more sensitive than physician examination or fundus imaging and detects capillary loss earlier than when the clinical lesions of DR become apparent (Hwang et al., 2015; Rosen et al., 2019; Schottenhamml et al., 2016; Yasin Alibhai et al., 2018), indicating its sensitivity and promise as a tool for assessing DMI (Cheung et al., 2021, 2022). However, it is worth noting that certain microvascular changes, such as microaneurysms associated with DR, may be detected more effectively with FA than OCTA, despite the latter’s ability to identify areas of nonperfusion (Schwartz et al., 2014). In addition, unlike FA, OCTA does not detect vascular leakage, an important feature of DR pathology. These considerations highlight the complementary nature of these imaging modalities in providing a comprehensive assessment of DR.

Here, we will discuss in detail several OCTA metrics that have potential for use in clinical trials of DR and its complications, how OCTA can assess anatomical changes resulting from DR and relate them to visual function, and how to interpret OCTA images, as well as the challenges and drawbacks of using OCTA in DR. We intend this article to be a definitive point of reference to guide future research using OCTA in DR, supporting the development of new treatments and improving the understanding of how DR can lead to vision loss.

## 4. Optical coherence tomography angiography metrics with potential for use in clinical trials of diabetic retinopathy, diabetic macular edema, and diabetic macular ischemia

### 4.1. Foveal avascular zone metrics

The FAZ is an area surrounding the fovea that is free of retinal capillaries, limiting light scattering within the foveal pit and resulting in enhanced vision quality (Chui et al., 2012; Shiihara et al., 2018). Loss or non-perfusion of the small capillaries at the margins of the FAZ is associated with reduced visual function (Endo et al., 2021). In this section, we will discuss using FAZ metrics in OCTA to stratify by disease severity and using change in FAZ metrics over time to evaluate for worsening disease.

FAZ size, whether measured by OCTA or by fluorescein angiography (FA), is negatively correlated with visual acuity (Balaratnasingam et al., 2016; Etdrs, 1991; Okumichi et al., 2021). In one study of 246 patients who had DMI, FAZ sizes were measured using FA and ranged from 0.27 to 0.78 mm^2^. The study found that patients with larger FAZ sizes, indicating more severe DMI, were significantly more likely to have decreased visual acuity (p < 0.001). Specifically, those with “moderate” or “severe” DMI, defined as FAZ ≥0.32 mm^2^, exhibited a greater likelihood of reduced visual acuity (Sim et al., 2013b). That said, despite numerous studies correlating DMI with vision loss (Balaratnasingam et al., 2016; Etdrs, 1991; Okumichi et al., 2021), the precise stage at which DMI affects visual function is unclear. Furthermore, other studies have shown a poor correlation between FAZ size and BCVA; this is confounded by the large physiological variability in FAZ size (discussed in more detail further on in this section). The lack of correlation between change in FAZ size and vision loss may be due to a time lag between enlargement and resultant vision loss or to the fact that a certain threshold of capillary dropout needs to be met before visual function declines. However, BCVA is not solely dependent on FAZ enlargement. It is also affected by other factors such as retinal neurodegeneration that results from diabetes itself, as well as associated inflammation. Concurrent pathologies in the macula, such as a history of DME or choroidal ischemia caused by diabetes, may also contribute to BCVA, further complicating the relationship between FAZ size and visual acuity. Establishing a threshold of FAZ size at which visual impairment occurs is difficult. Microperimetry used in conjunction with OCTA may be one option to detect impaired visual sensitivity resulting from macular ischemia at an earlier stage than BCVA (Tsai et al., 2020).

Increased FAZ size can be used as an indicator of DMI severity (Balaratnasingam et al., 2016; Hamid et al., 2020; Krawitz et al., 2018; Sim et al., 2013b), and FAZ size positively correlates with overall DR severity and progression (Mansour et al., 1993; Sun et al., 2019). In a study including 205 eyes, an increase in FAZ size significantly predicted the progression of DR (p < 0.001). In addition, the FAZ shows a higher degree of irregularity in patients with severe DME compared with those with earlier-stage DME (Custo Greig et al., 2020; Han et al., 2022). These data suggest that measurements of the FAZ may provide insight into the status and progression of DR, DME, and DMI (Hwang et al., 2016a). It is important to note that FAZ size can exhibit variability between the superficial and deep plexus and may vary with segmentation even within the same eye, depending on the OCTA machine used. This variability in FAZ size measurement presents an additional layer of complexity when assessing extent of DR. The ETDRS grading system is the gold standard for assessing DR severity and predicting DR progression (Mehta et al., 2021). Retrospective comparison of OCTA images demonstrated that superficial and deep-retinal OCTA slabs correlate significantly with ETDRS grades (p < 0.001) (Hwang et al., 2018; Mehta et al., 2021).

**Fig. 1.**
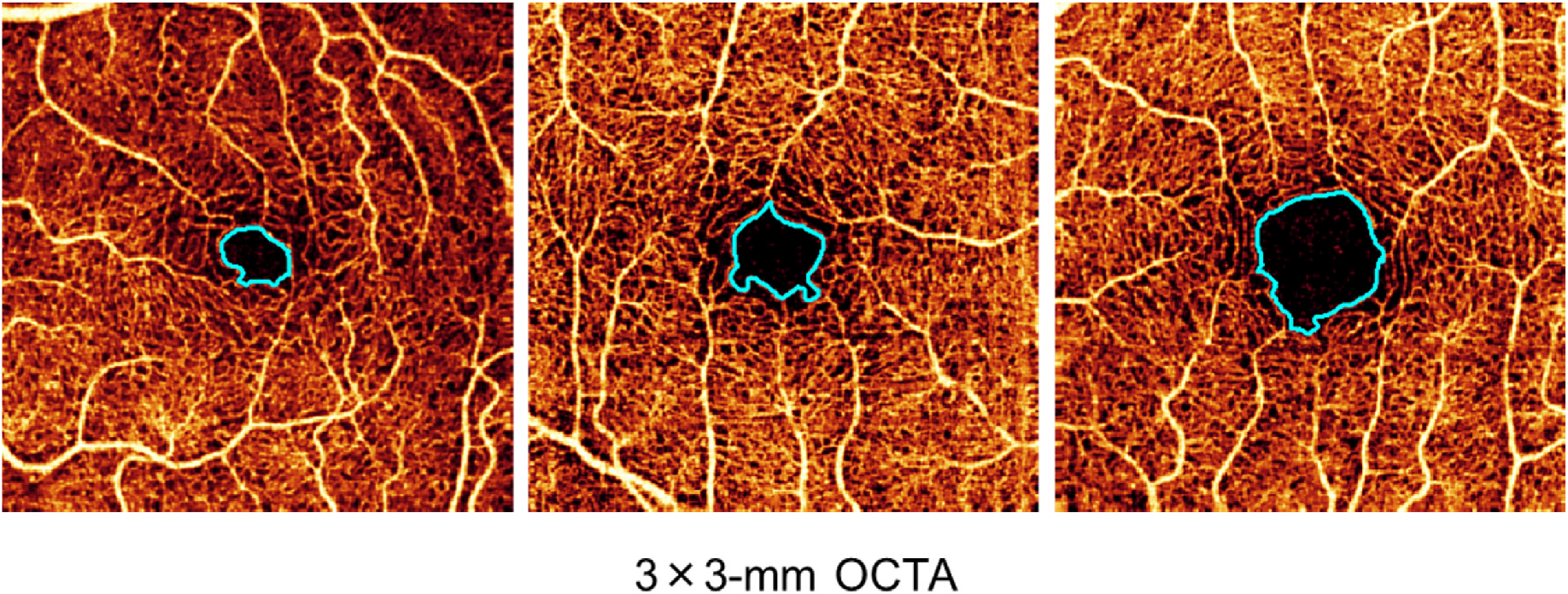
Variance of FAZ size (light blue outline) in three different healthy volunteers; left to right, FAZ size is 0.15 mm^2^, 0.20 mm^2^, and 0.44 mm^2^. The size of the FAZ is highly variable in healthy eyes. FAZ, foveal avascular zone; OCTA, optical coherence tomography angiography.

FAZ size can have high inter-person variability (up to ~3-fold; Fig. 1), limiting its utility for confirming DR-induced capillary nonperfusion (Wang et al., 2019) unless baseline images are taken before the onset of the pathology. Several studies have examined FAZ area in normal eyes, which can vary substantially between individuals (e.g., 0.110–0.861 mm^2^) (Eldaly et al., 2020; Shiihara et al., 2018). In addition, other pathological features, such as epiretinal membrane or the presence of center-involving DME, may alter the size and contour of the FAZ, complicating comparisons. Furthermore, the structure of the FAZ, composed of a convergence of multiple vessel complexes, complicates the quantification of border changes assessed using conventional two-dimensional imaging (Wang et al., 2019). Although it is difficult to establish an accurate baseline size from fundoscopy or FA, it is possible to compare an OCTA image with its matching OCT reflectance image to reveal the extent of an individual’s FAZ enlargement. This is achieved by comparing blood vessels between the *en face* reflectance and angiographic perfusion images, thus generating a differential image (Lynch et al., 2018).

There are only a limited number of studies examining change in FAZ size over time, but some data indicate that FAZ size and appearance can change significantly even between just two clinic visits (p < 0.05; mean visit interval 3.2 months) (Gill et al., 2017), though this could be due to differences in image segmentation or receipt of treatment by some patients between visits. FAZ size changed in eyes either with and without treatment (Gill et al., 2017), making it difficult to assess which changes were the result of an intervention, disease progression, or segmentation variability. In any case, because of the variability in FAZ size in the healthy populations, the use of FAZ area alone to stratify patients by DR severity is highly challenging, limiting its value both in the clinic and as a stratification factor for studies. Changes in FAZ size, however, may be a potential study endpoint.

An alternative measure to FAZ area size is FAZ circularity index (4*πA/P*^2^; where A is FAZ area and P is FAZ perimeter) (Kim et al., 2018). The normal shape of the FAZ approximates a circle, with a continuous loop of capillary around the perimeter. The FAZ is known to become more tortuous or acircular in patients with DR (Krawitz et al., 2017), as the dropout of capillaries at various locations increases the perimeter reducing its circularity. This suggests that FAZ circularity is a more sensitive metric in patients with DR than FAZ area alone. However, this also suggests that the FAZ circularity is more sensitive to factors associated with OCTA image acquisition variability than FAZ area. The inter-person variability of FAZ circularity is lower than that of FAZ area (Linderman et al., 2018), which may make circularity more suited for the assessment of DR severity than FAZ area when no longitudinal data are available. Often, patients with severe DME, which commonly occurs in severe DR, have greater FAZ acircularity (Han et al., 2022). Thus, the presence of DME may confound the utility of FAZ circularity to grade DR severity. That said, changes in FAZ parameters over time (including FAZ circularity) measured using OCTA may be useful as a meaningful parameter in clinical trials. Some work has shown that there are significant changes in FAZ circularity for patients with moderate-to-severe DR over a 1-year period, regardless of the presence of DME, but not in patients with no or mild DR (Tang et al., 2018). This suggests that changes in the FAZ over 12 months for patients with DR versus controls could be used to assess DR severity, with some evidence indicating that long-term changes in FAZ may be used to predict DR progression (Suzuki et al., 2018). Finally, it should be noted that, although the immediate repeatability of FAZ size measurement is high, with correlation coefficients >0.9, little is known about the repeatability over a longer period of time (Eastline et al., 2019).

**Fig. 2.**
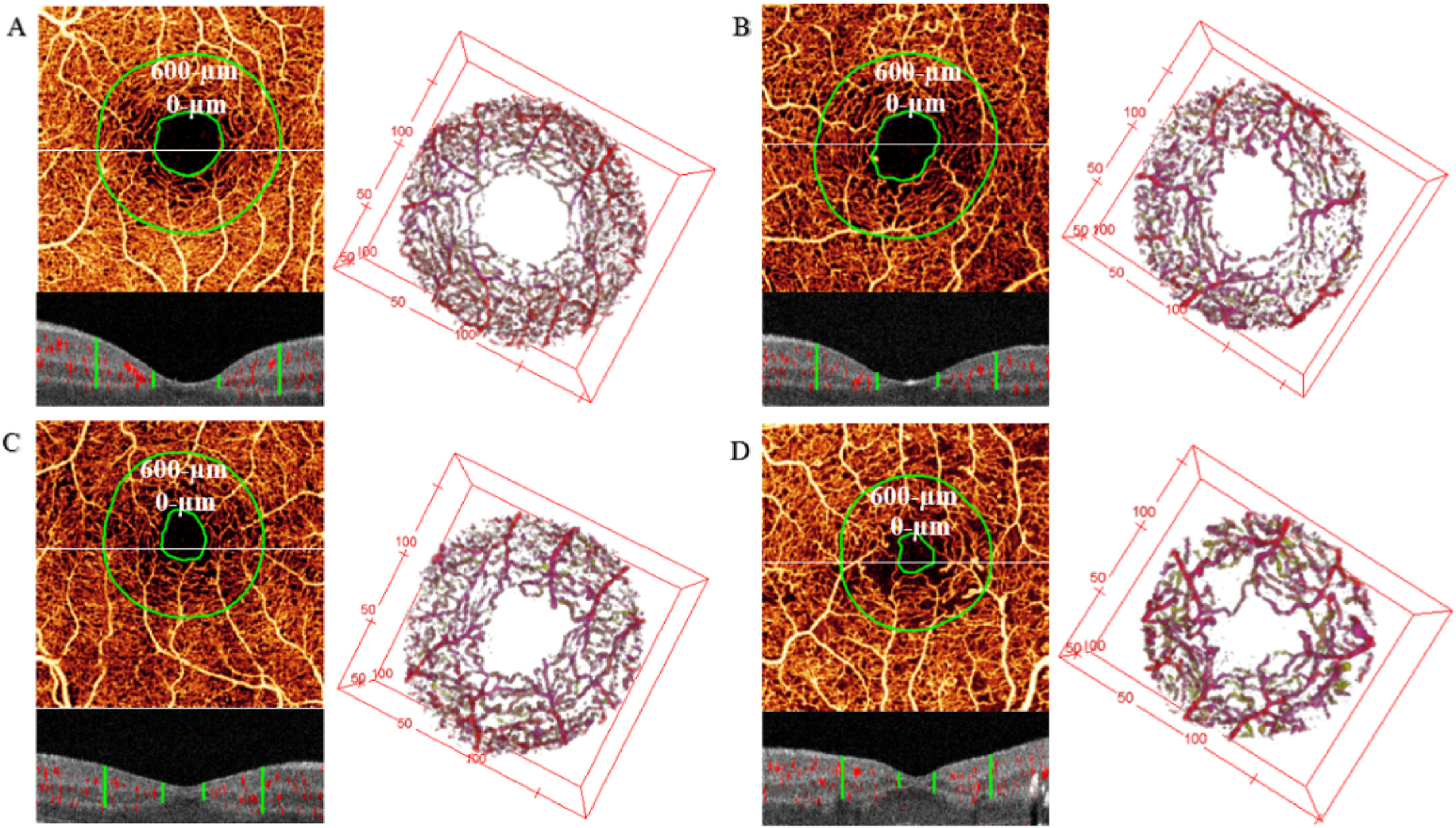
Three-dimensional para-FAZ vessel density in the eyes of a healthy control (A), with diabetes without retinopathy (B), with mild-to-moderate non-proliferative diabetic retinopathy (C), and with proliferative diabetic retinopathy (D). Upper left panels of (A–D): *en face* maximum projection of inner-retinal angiogram. The inner green line represents the tbFAZ boundary; the outer green line represents 600 μm distances from the tbFAZ boundary in the transverse direction. The white horizontal line indicates the position of the representative B-scan in the panel below. Lower left panels of (A–D): cross-sectional B-scan overlaid with angiographic signal (red). The green vertical lines indicate the analytic para-FAZ volume boundary locations in the inner retina. Right panels of (A–D): corresponding volumetric para-FAZ optical coherence tomography angiography. FAZ, foveal avascular area; tbFAZ, theoretical baseline FAZ. Reproduced with permission from Wang, B. et al., 2019. Biomed Opt Express 10, 3522.

Another alternative measure of FAZ-related macular ischemia may be 3D para-FAZ vessel density (Fig. 2) (Wang et al., 2019). This FAZ measurement is based on an estimate of FAZ size before the onset of DR, termed theoretical baseline FAZ (tbFAZ). The boundary of tbFAZ is defined based on the typical relationship between ganglion cell complex thickness (from structural OCT) and FAZ boundaries (from OCTA) in the normal population. With the addition of deep-learning-based macular fluid segmentation (Guo et al., 2020), tbFAZ can be estimated in eyes with DME. Estimation of tbFAZ is followed by the construction of a para-FAZ volume and calculation of projection artifact-resolved 3D vessel density (Wang et al., 2019). Compared with conventional FAZ measurements, 3D para-FAZ vessel density has demonstrated greater correlation with DR severity and higher diagnostic sensitivity in differentiating DR of various severities (Wang et al., 2019).

High-resolution OCT and OCTA volumes are required to develop robust parafoveal or para-FAZ ischemia metrics. With further improvement of resolution, OCTA may better reveal anatomical and vascular details, while also allowing the development of sensitive biomarkers. Ideally, FAZ should be assessed using a full-retinal slab to limit segmentation errors and confounding factors, such as center-involved DME. Furthermore, it is critical that all OCT images of the FAZ be checked not only for comorbid conditions, such as epiretinal membrane and DME, but also for low image quality, low resolution, artifacts, and segmentation errors. The inclusion of additional parameters when measuring FAZ area, such as 3D para-FAZ vessel density and FAZ circularity, would enrich the data and enhance the accuracy and insightfulness of the assessment of patients with DR.

### 4.2. Vessel density

The relatively low density of capillaries in the primate retina is the result of a balance between vascularization, to support the metabolic needs of visual information processing, and limitation of the number of blood vessels, to avoid hindrance of the passage of photons to the retina (Puro, 2012). This means that there is little capacity for a reduction in retinal vessel density without a direct impact on vision (Puro, 2012); therefore, the density of the retinal vascularity is a useful metric for estimating visual function in the macular area.

Vessel density is a quantitative metric that approximates the number of vessels occupying a particular region of the retina. This can be evaluated in various ways. It may be assessed as the proportion of vessel area with blood flow over the total measured area (vessel area density or flow density) or as the total vessel length in an area of interest (vessel length or skeletonized vessel density) (Pramil et al., 2021; You et al., 2019). As retinal vasculature is known to change with retinal pathology, measuring vessel density may provide insight into microvascular diseases like DR and, in particular, ischemic complications such as DMI (Pramil et al., 2021). Furthermore, unlike FAZ area, vessel density is not associated with age or sex, making it a potentially more reliable measure (Eldaly et al., 2020; Pramil et al., 2021; Shiihara et al., 2018).

There are a number of ways to measure vessel density, but the most common metrics are vessel area density and vessel length density (Pramil et al., 2021). Skeletonization is a process in which the width of each detected vessel is reduced to 1 pixel in size; following this, total vessel length is calculated. In theory, this reduces the impact of image resolution on the apparent vessel caliber (Chu et al., 2016), but images with low signal strength can lead to discontinuity in the skeletonized vascular network. There are advantages to both skeletonized and non-skeletonized methods. Skeletonization reduces the weighting of large blood vessels and may therefore increase sensitivity to microvascular changes. Conversely, non-skeletonized methods provide more physiologically relevant information about the overall vasculature as vessel size is accounted for (Al-Sheikh et al., 2017).

**Fig. 3.**
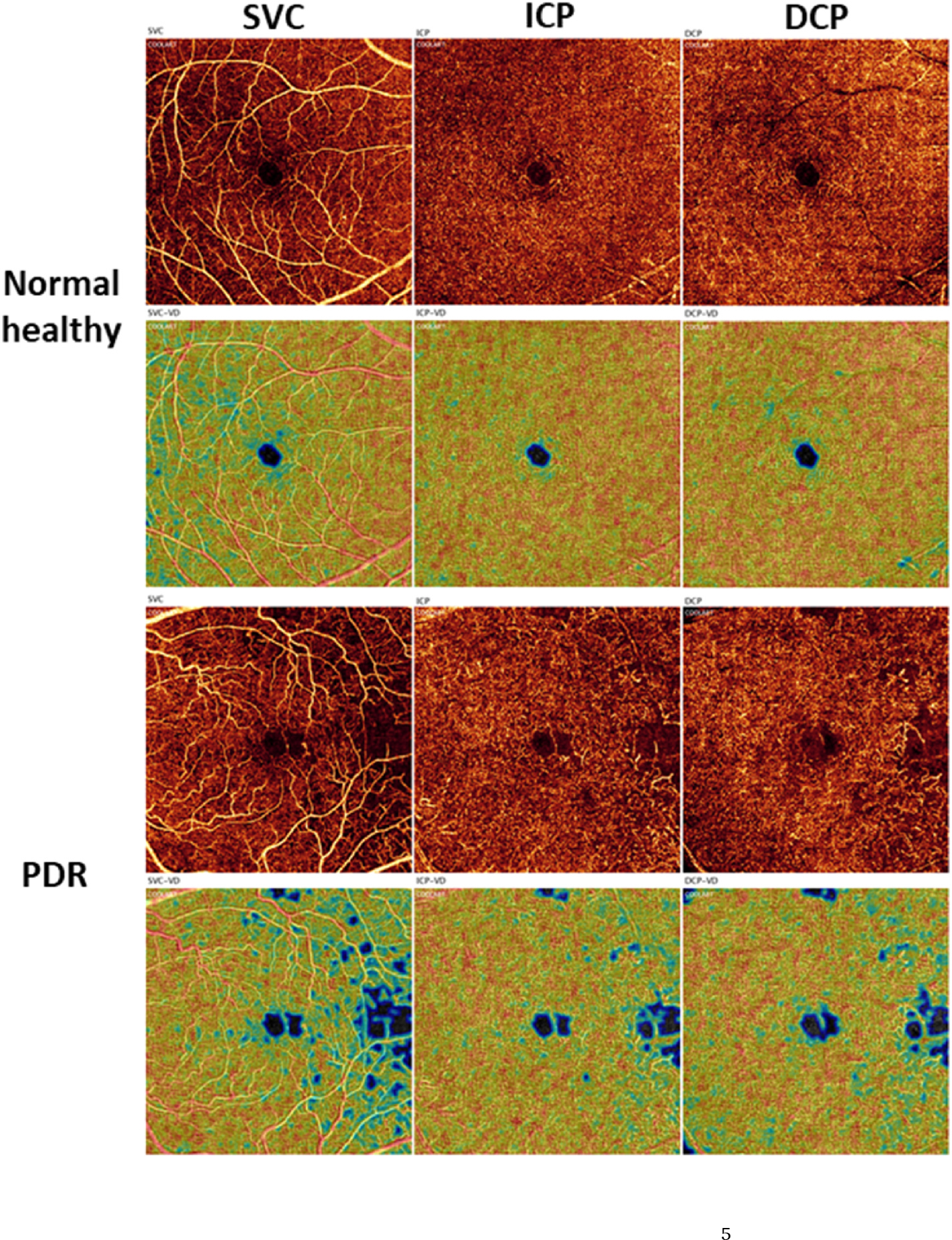
Projection-resolved 6 × 6-mm optical coherence tomography angiograms (1st and 3rd rows) and corresponding vessel density heat maps (2nd and 4th rows) of a healthy eye and an eye with PDR in the SVC (1st column), ICP (2nd column), and DCP (3rd column) (previously unpublished data; the published method can be found in (Hagag et al., 2019). Am J Ophthalmol 204, 70–79). DCP, deep capillary plexus; ICP, intermediate capillary plexus; PDR, proliferative diabetic retinopathy; SVC, superficial vascular complex.

Several studies have shown that decreased vessel density across the capillary plexuses correlates with increasing severity of nonproliferative diabetic retinopathy (NPDR) (Agemy et al., 2015; Hwang et al., 2018; Nesper et al., 2017; Ong and Fawzi, 2022; Ong et al., 2020; Onishi et al., 2018). In patients with PDR, there is likewise a clear reduction in vessel density across all capillary plexuses (Fig. 3, previously unpublished data; the published method can be found in (Hagag et al., 2019)). Interestingly, some research has shown that patients with diabetes but without DR have higher capillary density than non-diabetic controls, potentially as a response to increased metabolic demand (Onishi et al., 2018; Rosen et al., 2019). Taken together, these specific data suggest that a decrease in capillary density can indicate disease progression, while an increase in density may indicate disease onset. However, this remains an area of debate, as other work has shown an increase in capillary density upon progression from moderate to severe DR (Or et al., 2019), which has been hypothesized to be correlated with an increase in VEGF burden resulting in vessel dilation. More research is needed to establish the relationship between DR stage and capillary density, with the understanding that, in general, worsening disease is associated with capillary dropout and decrease in density.

Decreased capillary perfusion and vessel density, including in the SVC and DVC, are also specifically linked to ischemia (Abdelshafy and Abdelshafy, 2020; Bradley et al., 2016; de Barros Garcia et al., 2017; Gildea, 2019; Scarinci et al., 2019), disorganization of the retinal inner layers (DRIL) (Onishi et al., 2019; Pramil et al., 2021), and neurodegeneration visible as ganglion cell layer thinning. Furthermore, decreased vessel density in the SVC may be associated with the development of DME (Sun et al., 2019). Importantly, changes in SVC vessel density may be more reliable than measures in the DVC, as they are less susceptible to noise and projection artifacts, even when artifact-removal algorithms are used (Hwang et al., 2016b, 2018; Ong et al., 2020; Sun et al., 2019; Zhang et al., 2016b).

To quantify vessel density in intermediate and deep capillary plexuses, a reliable algorithm (Patel et al., 2018; Wang et al., 2017; Zhang et al., 2016a) should be applied to remove the projection artifacts. To generate a reliable vessel density map, it is also necessary to compensate for the effect of reflectance variation (Gao et al., 2016a) and remove any invalid areas caused by shadow artifacts (Camino et al., 2019b). Furthermore, if there is a strong bulk motion presence, inclusion of a preprocessing step to subtract the bulk motion (Camino et al., 2017, 2018) is a prerequisite. With the assistance of artificial intelligence, high-resolution capillary maps can be reconstructed and enhanced to facilitate reliable vessel density measurements, although this is very much an active work in progress. Using this technology (Gao et al., 2020, 2021), definite and clear capillary plexuses can be reconstructed from large field of view scans (e.g. 6 × 6-, 9 × 9- or 12 × 12-mm scans) that have been under-sampled.

One major issue in the reliability of vessel density metrics is the ability to accurately segment the individual capillary plexuses in eyes that have structural abnormalities related to DR, such as the presence of edema or ischemic-related structural changes. Therefore, the most repeatable and reproducible metric may be the evaluation of full-thickness retinal vessel density, which provides data for all retinal layers. This facilitates the alignment of longitudinal scans within patients and the distinction of each retinal layer even in the presence of pathology. Unfortunately, the inbuilt software of OCTA devices produced by some OCT manufacturers does not support the assessment of full-thickness retinal vessel density, which means that non-standardized custom software is typically required. Another factor of increasing importance when assessing vessel density using OCTA is the image field of view. Capillary density in the DVC decreases toward the periphery of the retina (Guo et al., 2020; Lavia et al., 2020). As OCTA technology has developed, a number of commercially available devices now offer wide-field and ultrawide-field OCTA (12 × 12 mm; 12 × 9 mm; 15 × 9 mm; 23 × 20 mm) (Hirano et al., 2020; Hong et al., 2020; Rosenfeld et al., 2016; Stanga et al., 2016; Yang et al., 2021). Research has also shown that OCTA metrics in the retinal periphery have high repeatability (Hong et al., 2020) and are therefore potentially more reliable between visits than other imaging methods. As wide-field technology becomes more commonplace, the assessment of peripheral vessel density may become a more important focus for research investigating DR.

Another metric, the intercapillary area, has been evaluated in patients with DR and may be more sensitive to vessel dropout than vessel density (Schottenhamml et al., 2016). However, it is also more dependent on image quality and therefore may show more variability across visits.

### 4.3. Measurements of flow speeds

Retinal vessel density and blood flow are closely related metrics. As with vessel density, the retinal blood supply operates within a narrow margin of error and must locally adjust for the high metabolic demands of the retinal neurons (Puro, 2012). Therefore, measures of blood flow can indicate when and where the retina is under metabolic stress or lacks sufficient perfusion to carry out its function. OCTA images indicate either the presence or possible absence of or slowing of blood flow. Changes in blood flow are central to the pathology of complications of retinal diseases, such as DME and DMI (Bradley et al., 2016; Kleinman et al., 2010) as well as microaneurysms (Nakao et al., 2018). The detection of microaneurysms using OCTA via adaptive optics scanning laser ophthalmoscopy (AOSLO) may be influenced by intramicroaneurysmal turbulence (Nakao et al., 2018). Recent studies highlighted the value of an *en face* image-averaging process in improving image quality and reducing background noise (Uji et al., 2017). This process has been found to enhance the visualization of microaneurysms with intermittent blood flow and increase the detection ratio in eyes with DR (Kaizu et al., 2020). In addition, utilizing extended interscan times allows for the detection of slower capillary flows that can enhance the detection of microaneurysms in OCTA scans (Kaizu et al., 2022). In terms of morphological assessment, there is a correlation between the morphological appearance of microaneurysms on OCTA and FA leakage (Schreur et al., 2018). However, a single OCTA scan may depict microaneurysms differently for each acquisition (Spaide et al., 2015). This variability can be mitigated by OCTA averaging, which has been shown to stabilize the visualization of retinal microvasculature. A recent analysis used these averaged OCTA images to demonstrate a correlation between microaneurysm morphology, DR stage, and FA leakage (Fukuda et al., 2022). One study suggested that OCT-identified microaneurysms may be able to be classified according to co-localized OCTA flow signal into active and inactive types. This study suggested that active microaneurysms are more likely to be associated with diabetic macular edema than those without flow (Gao et al., 2023).

Variable interscan time analysis (VISTA) produces maps of relative blood flow, coded by color, that can indicate where in a specific image blood flow is relatively altered (Arya et al., 2018). Although the technique does not yet provide absolute measures of flow, cannot quantify flow, and can be affected by shadowing projection artifacts and motion artifacts, the values retrieved are repeatable between scans (Arya et al., 2018). Recent work confirms that VISTA can be used to detect relative blood-flow speeds in microvascular changes associated with DR (Arya et al., 2021). Changes in adjusted flow index (the average decorrelation value of all pixels in the OCTA image that are above the noise threshold (Jia et al., 2015; Nesper et al., 2017)) in the capillary plexuses correlate with DR progression, with measurements at the DVC typically declining with increasing DR severity, while the SVC remains stable (Nesper et al., 2017; Ong et al., 2020). Interestingly, some OCTA data indicate that, following panretinal photocoagulation, capillary-layer flow metrics increase in patients with high-risk PDR (Fawzi et al., 2019). It is also important to note that the repeatability of vascular density measurements in the three plexus layers, especially the ICP and DCP, decreases with an increase in the size of the OCTA scan and the presence of macular edema (Mukkamala et al., 2021). A study using ultrahigh speed, swept-source optical coherence tomography (SS-OCT) angiography demonstrated the occurrence of retinal and choriocapillaris microvascular abnormalities in all stages of diabetic retinopathy, even in diabetic patients without retinopathy (Choi et al., 2017). An analysis of the association between DR severity and macular choriocapillaris flow deficit percentage found that choriocapillaris flow impairment corresponds to DR severity, with all regions of the choriocapillaris significantly affected (Dai et al., 2020). To evaluate and quantify this accurately, algorithms need to be developed that can distinguish choriocapillaris flow deficit from shadowing (Camino et al., 2019a; Gao et al., 2017). Overall, measurement of flow remains an emerging field that may show promise in patients with macular changes related to DR. However, these metrics are currently not available on commercial OCTA devices.

### 4.4. Extrafoveal avascular areas

**Fig. 4.**
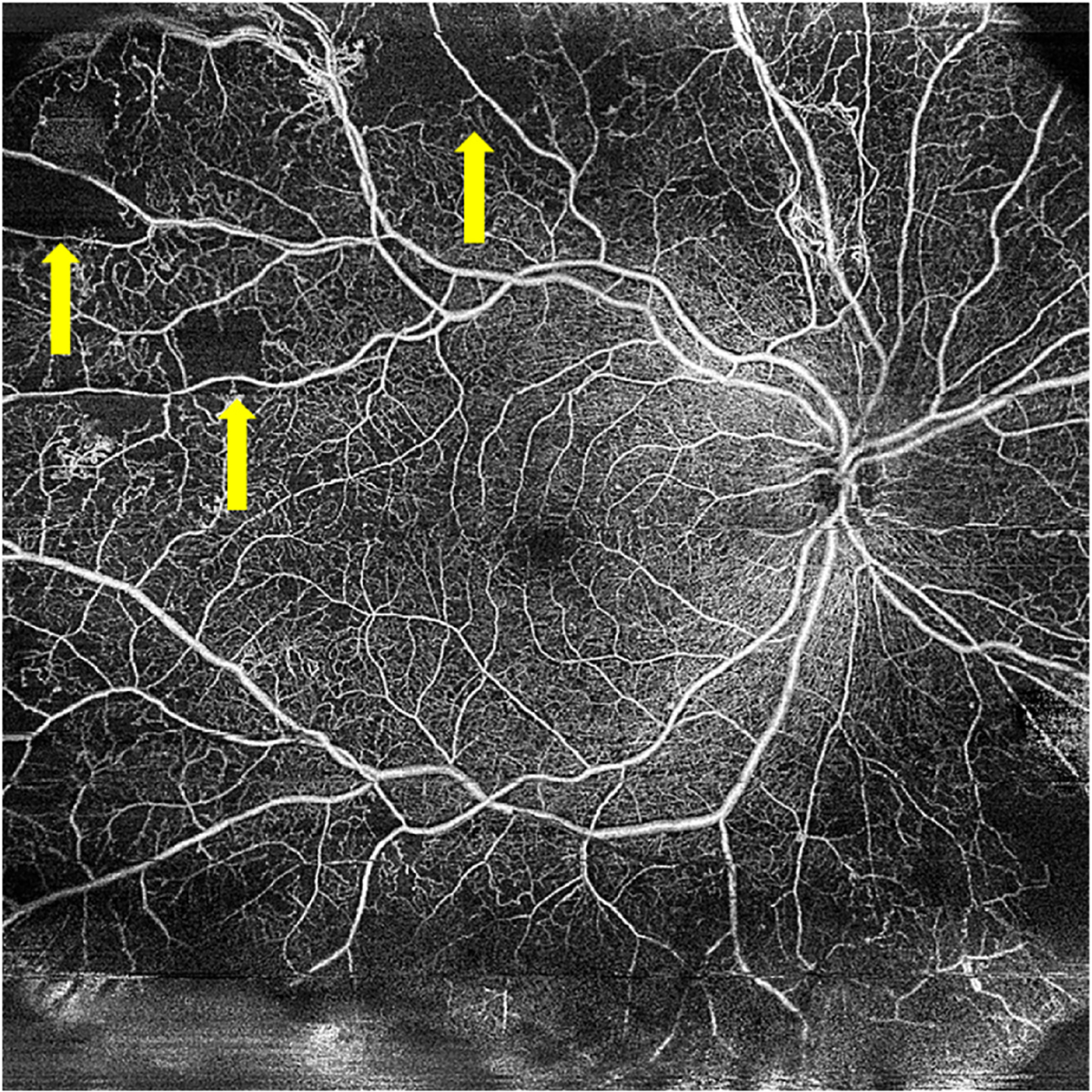
15 × 15 mm Optical coherence tomography angiography image showing areas of extrafoveal capillary dropout (yellow arrows), indicating extrafoveal avascular regions in a patient with proliferative diabetic retinopathy.

**Fig. 5.**
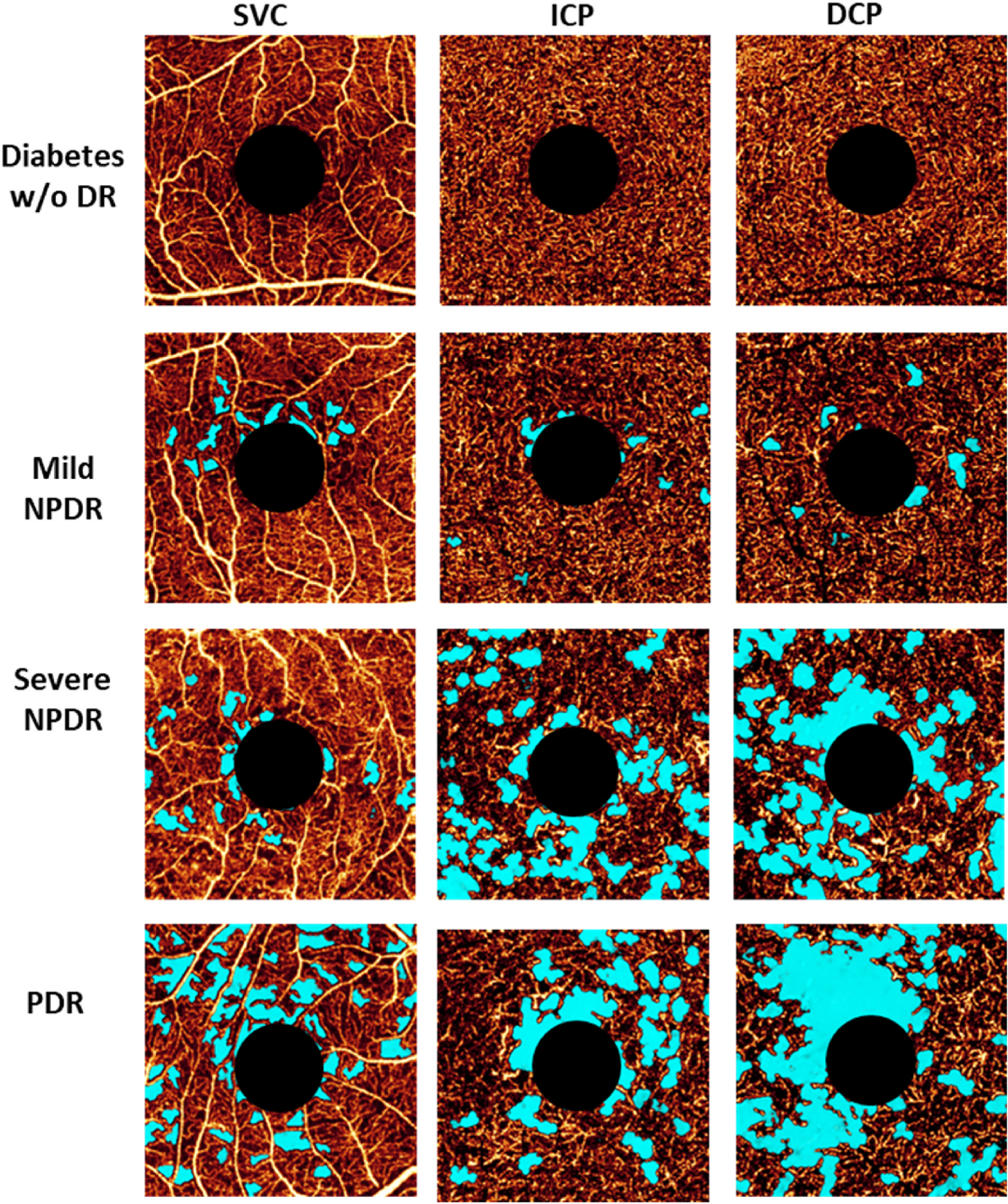
Presentation of the avascular area (light blue) in eyes with different DR severity. Images are projection-resolved 3 × 3-mm optical coherence tomography angiograms of a diabetic eye without retinopathy and of eyes with mild NPDR, severe NPDR, and PDR. The SVC, ICP, and DCP are presented in separate *en face* angiograms. DCP, deep capillary plexus; DR, diabetic retinopathy; ICP, intermediate capillary plexus; NPDR, non-proliferative diabetic retinopathy; PDR, proliferative diabetic retinopathy; SVC, superficial vascular complex; w/o, without. Adapted with permission from You et al., 2020a. Am J Ophthalmol 217, 268–277.

Extrafoveal avascular areas are defined as any region of avascularity outside the foveal region (e.g., Fig. 4) (Hwang et al., 2018; Schottenhamml et al., 2016; You et al., 2020b). Within the macula (Fig. 5), these are usually defined as being outside the central 1 mm circle of the SVC/DVC, which defines the fovea. Avascular areas outside the fovea but within the macula appear to be associated with risk of progression and vision loss (You et al., 2020b).

Detecting avascular areas has been traditionally done using FA, but leakage from the microvasculature can lead to inaccurate evaluations. OCTA, on the other hand, allows for the quantitative detection of non-perfused areas without the confounding effect of leakage (Nakao et al., 2018). Measurement of such areas is relatively straightforward and can provide a repeatable and reproducible automated metric for the evaluation of retinal ischemia (Hwang et al., 2018; You et al., 2020b; Zhang et al., 2016b). Compared with analyses within non-segmented slabs that comprise the entire inner retina, extrafoveal avascular areas in the macula can be better assessed by dividing the retina into three slabs: SVC, ICP, and DCP (Campbell et al., 2017; Hwang et al., 2016b) (Fig. 5). In addition, when avascular area is measured in three separate slabs, it is superior at detecting DR at different severities than the measurements from a commercially available vessel density in 2-layer scheme (Hwang et al., 2018). However, such metrics, while more sensitive, are also more dependent on image quality and therefore may be prone to issues with repeatability and reproducibility (Schottenhamml et al., 2016).

In patients with severe DR, areas of capillary dropout are clearly visible in OCTA images (Fig. 5) (Alibhai et al., 2020; Ishibazawa et al., 2019; Sorour et al., 2019; You et al., 2020b). Research has demonstrated that extrafoveal avascular areas detected using OCTA are significantly associated with DR severity (p < 0.0001), progression (p = 0.04), and treatment requirement (p = 0.002) over a 1-year period (You et al., 2020b). As more OCTA data on extrafoveal avascular areas both within and outside the macula are collected, it may be possible to collate a database of pathology-stratified extrafoveal avascular area measurements. This database could then be used to enhance the definitions of disease severity in DR.

Outside the macula, the retinal plexuses merge as the retina becomes thinner with greater eccentricity (Lavia et al., 2020), and so capillary dropout is more reliably visualized using *en face* OCTA of the entire inner-retinal slab. Wide-field OCTA montages capturing extrafoveal avascular areas outside the macula show that the size of these areas correlates with DR severity and visual acuity (Guo et al., 2021).

With wide-field OCTA becoming more accessible (Hong et al., 2020; Rosenfeld et al., 2016; Stanga et al., 2016; Yang et al., 2021), the assessment of extrafoveal avascular areas is likely to become a focus of research in DR. Areas of midperipheral non-perfusion may prove more sensitive than macular non-perfusion for stratifying and staging DR. Recent research examining deep-learning approaches (Hormel et al., 2021b) to distinguish avascular areas from signal-reduction artifacts have shown promise and can provide reliable measurements across a range of different scan qualities (Guo et al., 2018, 2019, 2021). However, although it is possible to analyze three layers of the macula with confidence, at present we are able to assess only the superficial layer of the periphery, making it difficult to evaluate the relative sensitivity of intra- and extramacular regions.

### 4.5. Intraretinal microvascular abnormalities

**Fig. 6.**
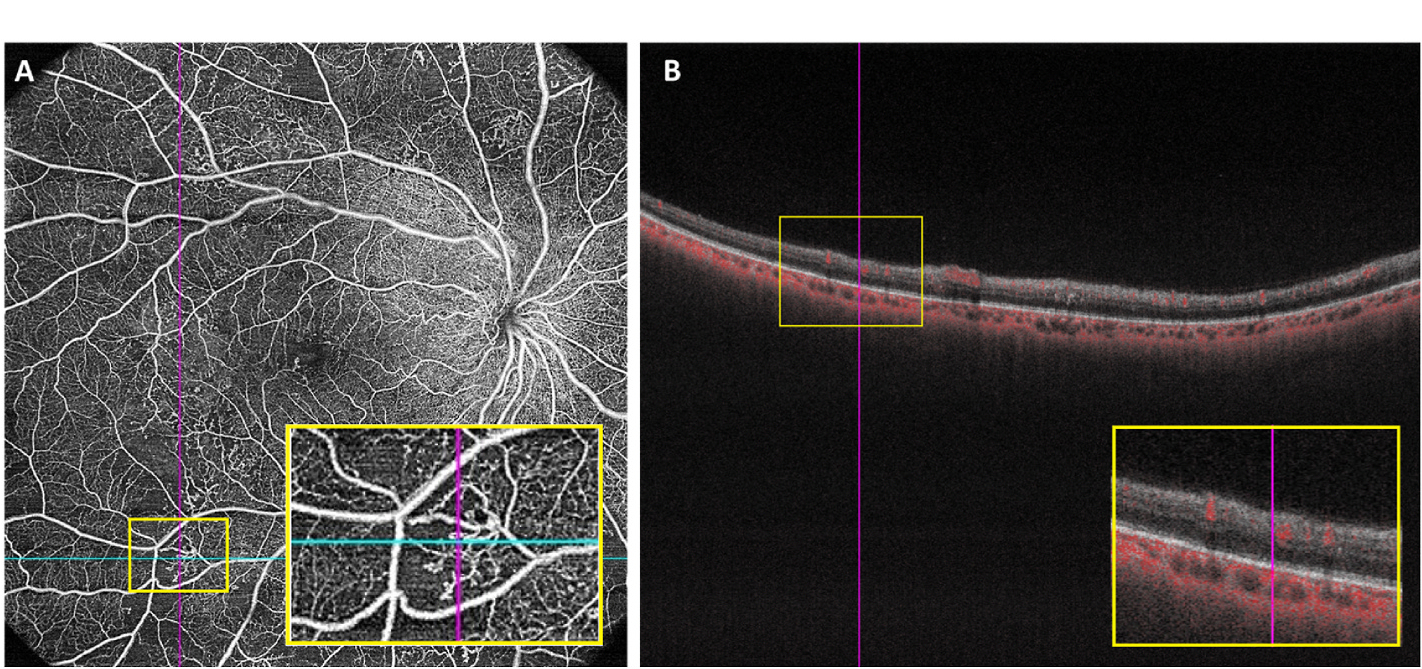
Example of IRMAs in one patient with proliferative diabetic retinopathy.(A) 15 × 15 mm Optical coherence tomography angiography image showing IRMA in a full-retinal slab, highlighted in an inset yellow box; (B) B-scan with segmentation lines in the same patient, taken as a cross-section from the blue line in (A), with intraretinal flow highlighted in the inset yellow box. The ILM and hyaloid face are not breached, thus differentiating the IRMA from neovascularization. ILM, inner limiting membrane; IRMA, intraretinal microvascular abnormality.

IRMAs are “torturous intraretinal vascular segments” (Fig. 6) (Etdrs, 1981) that vary in size and can be almost undetectable (Shimouchi et al., 2020). They are associated with severe NPDR (ETDRS Group, 1991). Therefore, the classification and detection of IRMAs may provide insight into the disease status of patients with DR.

Results from a secondary analysis of the CLARITY study using widefield fundus imaging suggested that IRMAs are improved by intravitreal anti-VEGF (Pearce et al., 2020). However, this treatment response will need further validation using OCTA. On *en face* OCTA images, IRMAs present as dilated, irregularly structured vessels, surrounded by capillary dropout (Sorour et al., 2020). OCTA is as sensitive as FA and more sensitive than color fundus imaging in detecting the features of IRMA; approximately 50% more IRMAs are detected on OCTA than on color fundus imaging (Hwang et al., 2015; Ishibazawa et al., 2015; Schaal et al., 2019). Furthermore, it is now possible to see differently shaped IRMAs using OCTA (Sorour et al., 2020). Importantly, as a 3D imaging method, OCTA allows accurate layer-specific localization of IRMAs within the retina, improving their differentiation from neovascularization, which would be detected on the retinal surface and breaking through the internal limiting membrane (de Carlo et al., 2016).

### 4.6. Neovascularization

Pathological neovascularization is a hallmark of PDR and can be examined using OCTA; treatment-naïve patients with PDR typically have irregular proliferation of fine new vessels, and this can be used as an imaging marker for active neovascularization (Ishibazawa et al., 2016). The detection rate of neovascularization of the disc (NVD) and neovascularization “elsewhere in the retina” (NVE; classed as neovascularization occurring outside the optic disc) by OCTA is superior to color photos and FA (Khalid et al., 2021; Pichi et al., 2020). An OCTA-based classification of NVD into types 1–4 has been established: however, types 1 and 2 are clinically undetectable (Khalid et al., 2021). NVE can also be examined in patients with DR using wide-field OCTA (Feng et al., 2021; Shiraki et al., 2022). Subclinical or suspected NVE can be detected by wide-field OCTA either by using a high-speed system or a montaged wide field of view from commercial OCTA devices (You et al., 2020a), suggesting that OCTA may improve the clinical evaluation of DR.

One study found that an increase in NVE is significantly associated with the degree of ischemia (p =0.009); furthermore, the type of growth pattern of NVE (“round” vs “ramified”) correlates with ischemia, with round growth resulting in a larger ischemic index than that for ramified growth (Shiraki et al., 2022). In addition, NVE measured by OCTA has been used to assess treatment response in patients with PDR (Feng et al., 2021). One limitation to the assessment of NVE by OCTA may be the field of view; even montaged OCTA scans do not consistently capture peripheral lesions. That said, the assessment of neovascularization patterns using OCTA can provide insight into not only the degree of pathological neovascularization in patients with DR but also the development of ischemia.

### 4.7. Neurovascular coupling

Neurovascular coupling refers to changes in blood flow in response to localized neuronal activity. As OCTA reflects retinal hemodynamics, it may be a useful tool for assessing neurovascular coupling in the retina (Kwan et al., 2020; Nesper et al., 2019; Wei et al., 2013; Zhang et al., 2020). Induced hyperglycemia has been shown to disrupt neurovascular coupling in healthy eyes (Kwan et al., 2020); the evaluation of the status of neurovascular coupling in the retina could therefore provide insight into the degree of metabolic disturbance as a measure of diabetic-induced dysfunction.

**Fig. 7.**
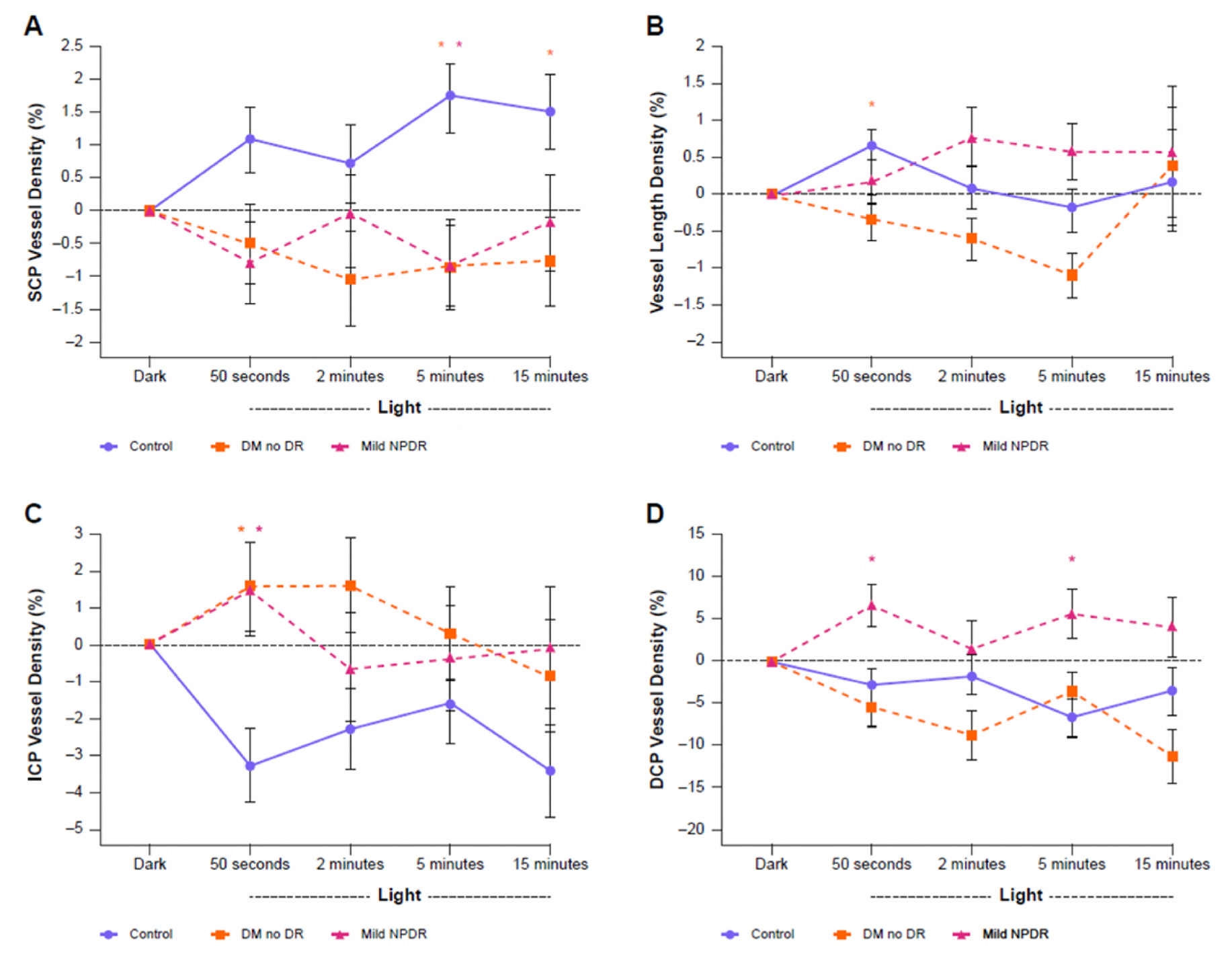
Parafoveal vessel density measured using OCTA at different retinal plexuses during ambient light transition for controls, patients with diabetes but no DR, and patients with mild NPDR. (A) Vessel density in the SCP; (B) vessel length density; (C) vessel density in the middle capillary plexus; (D) vessel density in the deep capillary plexus. Statistically significant differences between diabetic and control conditions (p < 0.05) are indicated with an asterisk (*). DCP, deep capillary plexus; DM, diabetes mellitus; DR, diabetic retinopathy; ICP, intermediate capillary plexus; NPDR, non-proliferative diabetic retinopathy; OCTA, optical coherence tomography angiography; SCP, superficial capillary plexus. Adapted from Zhang, Y.S. et al., 2020. J Clin Med 9, 3523.

In 2020, OCTA was used to examine the extent of neurovascular coupling impairment present in patients with diabetes prior to the onset of visible anatomical changes associated with DR. In patients with diabetes without anatomical evidence of DR, altered neurovascular responses were similar to those recorded in eyes with mild NPDR (Fig. 7) (Zhang et al., 2020). This suggests that changes in neurovascular responses assessed using OCTA could detect changes that precipitate DR before the onset of identifiable structural alterations.

## 5. Relating optical coherence tomography angiography metrics to parameters of visual function

### 5.1. Best-corrected visual acuity

BCVA is a commonly used, regulator-accepted clinical trial endpoint of visual function (Cheung et al., 2021). Establishing anatomical metrics that correlate with BCVA could be useful as an objective surrogate endpoint for clinical trials as well as a proxy for predicting changes in vision, especially those that are not measured with conventional visual function testing methods. Although there are limited data examining the correlation of OCTA metrics with visual acuity, preliminary data indicate that there may be potential avenues worth further exploring, although some of the data are conflicting. In one study, OCTA 3D para-FAZ measurements were shown to significantly correlate with measures of BCVA (p < 0.01), as opposed to measurements such as FAZ area and perimeter, which in some studies do not (Wang et al., 2019). In another study, combining FAZ area with FAZ shape irregularity was shown to correlate with both DR grade and BCVA (Lu et al., 2018). Large baseline FAZ areas in the DVC revealed by OCTA have also been shown in one study to be predictive of worsening visual outcomes over the course of a year in patients with diabetes (Tsai et al., 2021). Interestingly, a stronger correlation was found between visual acuity loss and changes in the vascular density of the deep plexus as compared to the superficial or intermediate plexus in eyes with DR (Dupas et al., 2018; You et al., 2020b). This suggests that different plexus layers might have different impacts on visual acuity loss in DR. Further research examining the correlation of OCTA with measures of visual acuity is necessary to validate the relationship.

### 5.2. Other possible metrics

DR is also associated with a reduction in contrast sensitivity (Stavrou and Wood, 2003). Some data suggest that contrast sensitivity may be a more appropriate measure for tracking progressive vision loss than traditional visual acuity metrics (Della Sala et al., 1985; Dorr et al., 2013). This suggests that an imaging measure that correlates with contrast sensitivity could provide more useful information than visual acuity alone.

Microperimetry is a method that can measure the retinal function of specific locations in the retina (Acton and Greenstein, 2013; Pereira et al., 2019; Scarinci et al., 2019; Tsai et al., 2020; Usman, 2018). Given the similarity of *en face* microperimetry and OCTA, the two measures could be overlaid to look for relationships between vascular features and functional performance. To date, it is unclear whether there is a correlation between OCT metrics and microperimetry outputs in patients with diabetic eye disease (Sachdev et al., 2019); recent work identified a correlation between OCTA-assessed vessel density (but not FAZ area) and microperimetry-assessed retinal sensitivity in eyes with advanced DR, but not in eyes with lower grades of severity (Levine et al., 2022) and in eyes with retinitis pigmentosa (Hagag et al., 2019). By using microperimetry and OCTA quantitative flow density, Arima et al. showed that vascular hyperpermeability may inhibit the retinal sensitivity reduction in the non-edematous ischemic diabetic retina (Arima et al., 2021). These findings highlight the potential value of combining these innovative tools for a more comprehensive understanding of the functional and structural implications of diabetic eye diseases.

## 6. Clinical trials using optical coherence tomography angiography metrics

Despite OCTA being a relatively recent innovation, a number of clinical trials have begun to incorporate OCTA metrics into assessments of DR as primary endpoints (Table 1). Additionally, many large ongoing trials in DR and DME have incorporated OCTA metrics as exploratory endpoints, including FAZ size, vessel density, and vessel perfusion; although this highlights the increasing interest in OCTA, it should be noted that it is often not mandatory for the sites to capture exploratory endpoint images, resulting in fewer data overall.

Change in FAZ size is one of the most commonly selected OCTA-assessed measurements of DR progression (Table 1). Vessel density and perfusion have begun to gain traction as metrics and clinical trial endpoints to assess retinal disease progression (Table 1). As most of these trials are currently ongoing, the results are not yet available; however, when the data are released, they may reveal how useful the OCTA is in clinical studies (Greig et al., 2020).

One potential issue with OCTA images taken during clinical trials is the degree of unevaluable data. One non-interventional study found that 53.5% of OCTA scans in clinical trials contain severe artifacts, substantially affecting the reliability of any quantitative assessment (Holmen et al., 2020), while an analysis of three multicenter clinical studies of patients with DR found that 39% of scans lacked the required signal strength index to be considered useable images (Lujan et al., 2021). However, another study found that over 90% of images obtained in a clinical trial setting were evaluable (Custo Greig et al., 2020). Clinical trials in ophthalmology may often operate under sub-optimal retinal imaging conditions, as enrolled patients may have vision loss that leaves them unable to fixate or otherwise follow ideal protocols, resulting in poor-quality images (Cheung et al., 2021). It is therefore critical that clinical trials are carefully planned to account for this during data collection and that additional time is allowed to assess OCTA quality and to re-image where necessary. In contrast to the findings of Lujan et al. and Holmen et al. the percentage of OCTAs that were excluded from the TIME-2b study was low; quality metrics for OCTA images were pre-stipulated and carefully monitored over time (Custo Greig et al., 2020).

## 7. Challenges using optical coherence tomography angiography in diabetic retinopathy, diabetic macular edema, and diabetic macular ischemia

### 7.1. Variability of anatomical measures between patients

Although FAZ measurements are relatively robust within individual patients, as discussed earlier there is considerable variability at the population level (Fig. 1) (Wang et al., 2019). Some evidence suggests that this variability can be overcome by using metrics such as 3D para-FAZ vessel density (Wang et al., 2019).

### 7.2. Variability of optical coherence tomography angiography metrics among optical coherence tomography angiography devices

Using different OCTA devices can introduce considerable variability in results from quantitative metrics. While intra-individual repeatability is quite high when using the same device, there is generally only moderate-to-poor agreement for OCTA metrics between different device models (Levine et al., 2020; Li et al., 2018). In one study, the repeatability of peripapillary capillary quantification differed significantly between four different OCTA device models (Spectralis, Optovue, Cirrus, and Triton) (p < 0.001) (Lei et al., 2018), and significant differences in the evaluation of vessel visibility (p < 0.001) and motion artifacts (p < 0.001) have been found in a separate study examining the AngioVue (Optovue), AngioPlex (Zeiss), AngioScan (Nidek), and Spectralis (Heidelberg Engineering) (both 3 × 3- and 6 × 6-mm scans) (Li et al., 2018). Choice of commercial OCTA instruments should be carefully considered to ensure that the approach and format of vascular density and FAZ measurements is appropriate to the needs of clinicians. This is mainly due to the varying methodologies employed by different devices for segmenting the retinal plexus layers and detecting vascular flow on OCTA. Each commercial device utilizes a unique software and algorithm, leading to potential variations in the output measurements. That said, one study found no significant difference in measured vessel density between four OCTA device models (Zeiss, Optovue, Topcon, and Heidelberg; p = 0.2) (Munk et al., 2017).

Until more definitive comparisons between OCTA devices are performed, it is difficult to definitively say which devices are the most sensitive or which are able to provide the most accurate measures of disease pathology. The data reported to date suggest that the choice of device model strongly affects the resulting OCTA metrics and image quality and may affect clinical trials in which different devices are used across different centers.

### 7.3. Image artifacts

**Fig. 8.**
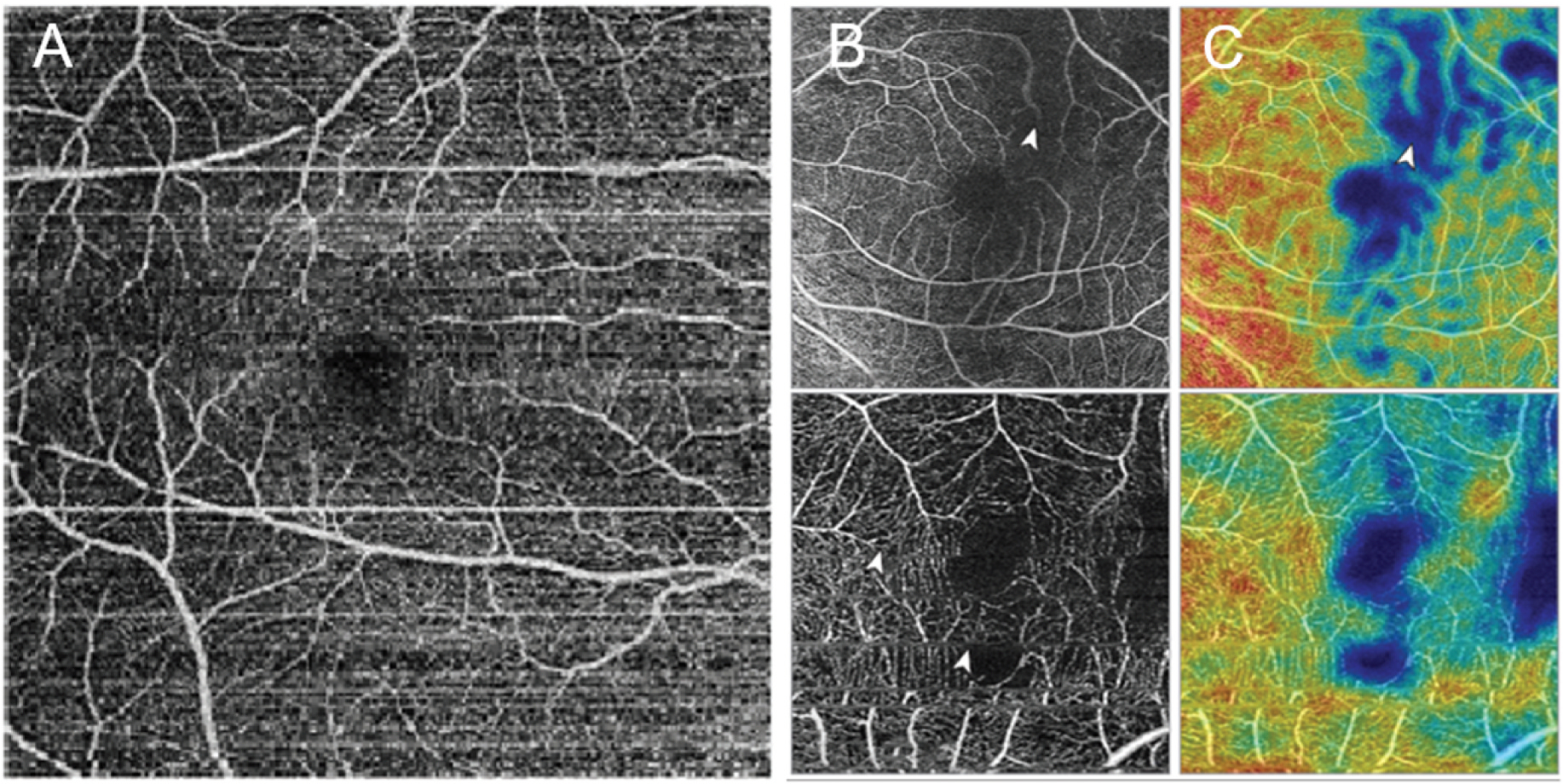
(A) Example of bulk motion artifacts in the superficial vascular complex from an AngioVue (Optovue, USA) optical coherence tomography device. (B) Optical coherence tomography angiography images with vascular marking: shadow artifact (top) and eye movement artifact (bottom). (C) Vascular density map of the same images as in (B): shadow artifact (top) and eye movement artifact (bottom). White arrowheads in B and C indicate movement lines. Adapted from Hormel, Teo et al., 2021. Quant Imaging Med Surg 11(3), 1120–1133.

**Fig. 9.**
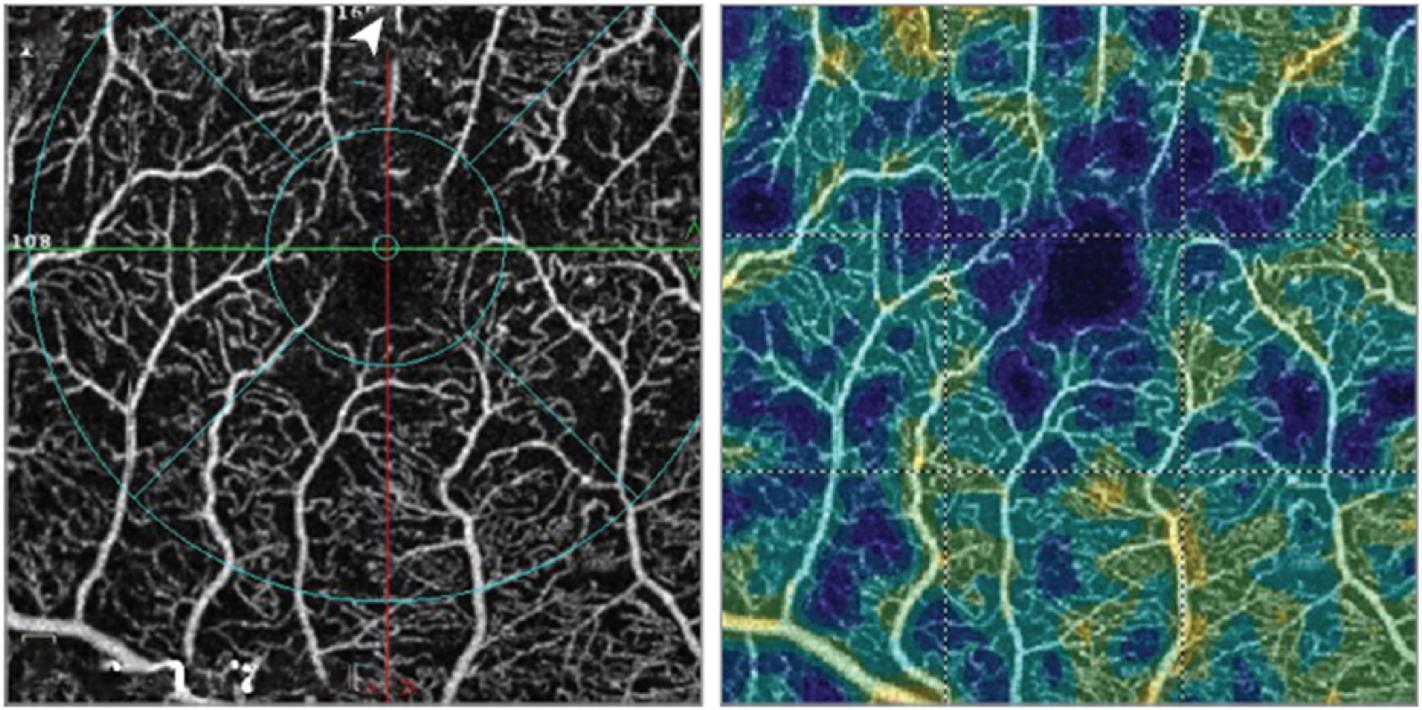
Example of a scan decentration artifact: optical coherence tomography angiography image (left) and vascular density map (right). Superior shift of the fovea when centering the scan results in the loss of the inner subfield (indicated by the white arrowhead). Adapted from Hormel, Teo et al., 2021. Quant Imaging Med Surg 11(3), 1120–1133.

Visualization of the retinal vasculature plexuses in 3D is one of the main advantages of OCTA. However, artifacts can cause misleading results (Hwang et al., 2016a, 2018); for example, motion artifacts caused by eye movement can result in the false appearance of decreased vascular density or “repetition” of anatomical landmarks, such as the fovea (Fig. 8 (Hormel et al., 2021a)). Common DR comorbidities, such as DME, DMI, epiretinal membrane, and vitreomacular traction, often overlap, resulting in vessel misidentification, segmentation errors, and noise artifacts in OCTA (Bazvand and Ghassemi, 2020). Patient movements causing bulk motion artifacts or failure of the operator to focus and center the scan can further affect image clarity (Fig. 9), hampering retinal vasculature identification and reducing the range of data captured (Bhende et al., 2018; Gao et al., 2016b; Hormel et al., 2021a; Onishi and Fawzi, 2019). Regardless of imaging system or scan protocol, the most common severe OCTA artifacts present in clinical trial data (406 images from 234 eyes) are shadow (26.9%), defocus (20.9%), and eye movement (16.0%) (Gao et al., 2021; Holmen et al., 2020).

Use of full-vascular-layer OCT removes the risk of projection artifacts, which can occur when the vasculature of more superficial layers project onto deeper layers during imaging. Projection artifacts can limit the accuracy of deeper-layer measurements; one research group has used capillary density-deviation mapping and a normative reference group to show that there is a significant reduction in parafoveal capillary density in patients with retinal pathology (including DR) versus healthy controls (Andrade Romo et al., 2019). However, as full-layer processing that incorporates both the superficial and deep microvascular layers, it does not allow the separate assessments of capillary dropout (avascular area) in individual vascular plexuses. Projection-resolved (PR)-OCTA offers a solution to address the deep-layer image artifacts without the limitations of full-layer OCT scans. PR-OCTA presents all three retinal vascular plexuses distinctly, increasing the accuracy of the visualization of deeper-layer capillary abnormalities that may otherwise be obscured (Hwang et al., 2018). In addition, algorithmic removal of decorrelation noise due to bulk motion in OCTA imaging appeared useful for improving the signal-to-noise ratio, contrast, vessel density repeatability, and noise in the FAZ (Camino et al., 2017).

**Fig. 10.**
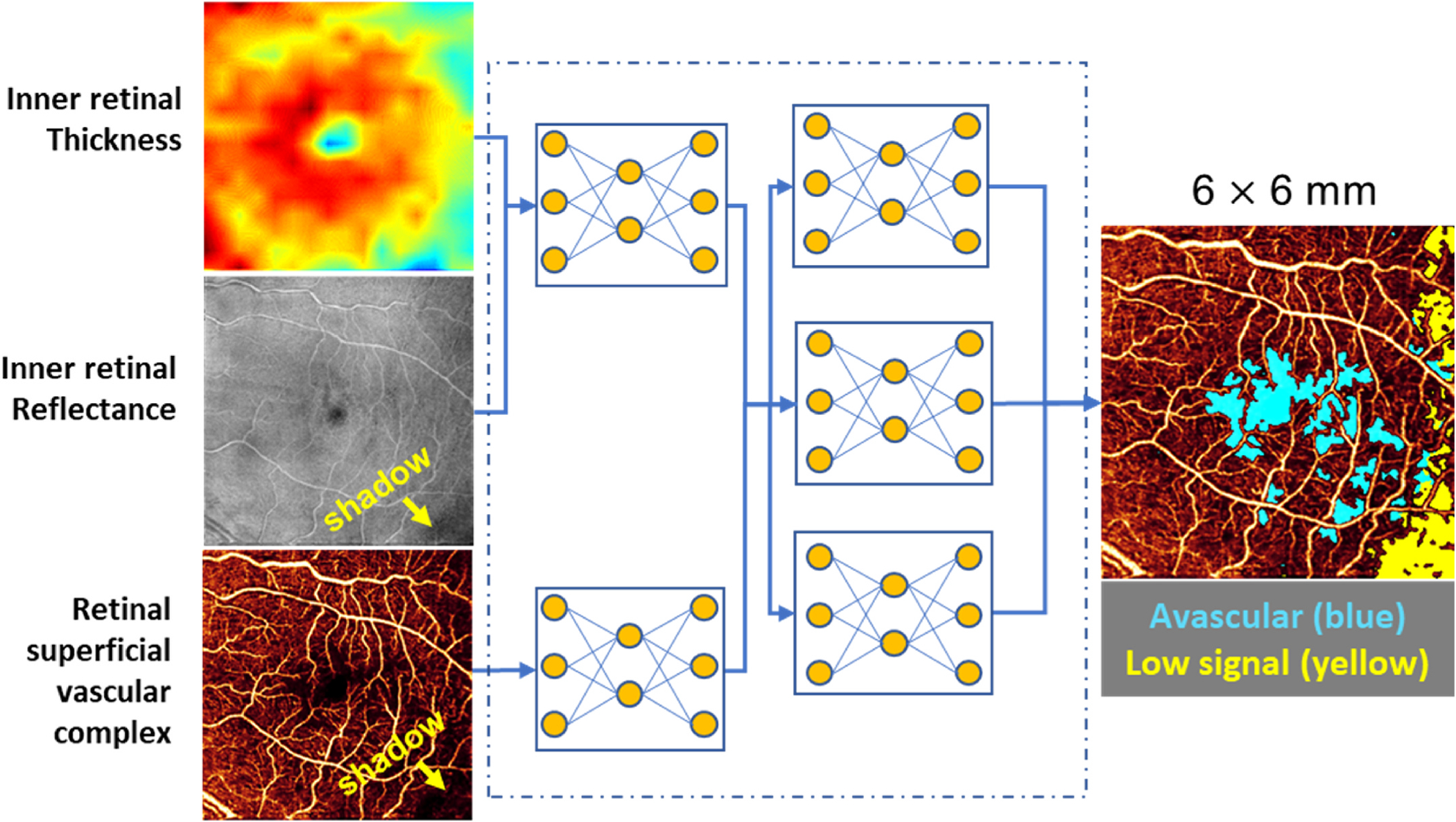
Algorithm for distinguishing non-perfusion areas from signal-reduction artifacts on OCTA. With an intelligent combination of structural OCT and OCTA data as the input, the convolutional neural network developed by Guo et al. can accurately distinguish between the real avascular area (blue) and shadow artifacts (yellow). OCT, optical coherence tomography; OCTA, optical coherence tomography angiography. Adapted from Guo, Y. et al., 2019. Biomed Opt Express 10 (7), 3257–3268.

In low-quality scans that have confounding signal attenuation due to shadow or defocus, low-signal areas can appear quite similar to avascular areas and are challenging to differentiate. Guo et al. have demonstrated a deep-learning algorithm (Fig. 10) that can automatically differentiate avascular areas from shadow artifacts (Guo et al., 2019).

Several OCTA artifacts and image distortions come from operator error; it is therefore critical to have an experienced and well-trained operator to minimize the incursion of artifacts at the point of data collection. Consistent protocols for imaging patients, including counting aloud the duration for which a participant must keep their eyes open, explaining where the patient must look, and carefully adjusting positioning equipment (such as chin rests) can ensure that bulk motion and blink artifacts are reduced.

After data have been collected, the standard procedure should include projection artifact removal to allow the clean separation of retinal plexuses. By viewing retinal plexuses separately, non-perfusion areas (capillary dropout) can be recognized more accurately than otherwise possible. It is also useful to view *en face* structural OCT images together with the corresponding OCTA images so that areas of shadowing (low OCT signal) can be recognized and distinguished from nonperfusion.

### 7.4. Lack of data

Current limitations in understanding the pathophysiology of DMI have hampered our ability to determine the predictive metrics for anticipating the progression of DMI (Cheung et al., 2021). That said, some recent research has used a normative database of OCTA data to develop a parafoveal capillary density-deviation mapping technique (Andrade Romo et al., 2019).

A recent Delphi consensus effort aimed to standardize OCTA nomenclature around retinal vascular diseases, specifically focusing on OCTA parameter definition and the assessment of DMI severity (Munk et al., 2021, 2022). Unfortunately, experts were not able to agree on a single metric in either the initial survey or the subsequent Delphi rounds; terms used to describe OCTA continue to vary notably between individuals (Munk et al., 2021). There remains a pressing need for more data and more consensus around OCTA in DR.

## 8. Future directions

### 8.1. Establishing a consensus and validating optical coherence tomography angiography metrics for diabetic retinopathy, diabetic macular edema, and diabetic macular ischemia

In order for OCTA to have true clinical utility, it is critical to establish a consensus among experts and to validate OCTA metrics through research and clinical trials for its use in DR (Fawzi, 2017). Similar to the endpoints used in geographic atrophy for pivotal clinical trials, anatomical endpoints have many advantages over functional endpoints. The first step is for regulators to accept that OCTA, as a measurement of blood flow, is a surrogate for retinal function. FAZ area and vessel density are clear markers of DR progression, but so far there are not enough data to correlate OCTA metrics with standard measures of visual function, such as visual acuity. Therefore, they are not yet acceptable as surrogate markers of visual function by regulatory authorities. Studies that validate the correlation between OCTA metrics and functional measures of vision will broaden the use of OCTA in research and clinical settings. The ultimate goal is to establish an anatomical endpoint based on OCTA that can be used in pivotal trials of new therapies.

### 8.2. Development of optical coherence tomography angiography technology and software to improve the imaging of diabetic retinopathy and its complications

As OCTA remains a relatively novel technology, it is important that there is continued research focus on improving image quality, particularly in the presence of common complications of DR that can confound clear measurements, such as DME. At present, standardized commercial software specifically for the imaging of DR or for OCTA in general that can address these issues is yet to emerge. However, the standardization of analysis algorithms between machines and research centers will facilitate data sharing and enable comparisons to be made between techniques and analyses. A 1-day workshop promoting the adoption of ocular imaging standards among the National Eye Institute (NEI), US Food and Drug Administration (FDA), and Office of National Coordinator for Health Information Technology (ONC) was recently conducted, which highlights the current focus on this topic (NEI-FDA-ONC, 2022). Automated image grading and automated image quality checks may address some concerns about the use of OCTA in clinical settings, reducing the need for specialized individual training while also providing a standard set of recognizable parameters for disease assessment.

## 9. Summary and conclusions

DR is a microvasculopathy, and OCTA signal intensity is tied to blood flow; theoretically, this positions OCTA as the ideal method for assessing DR. An increasing body of research indicates that OCTA can be used to quantitatively assess DR and its complications, even before the onset of clinical lesions. Several features characteristic of DR pathology can be assessed using OCTA, including FAZ size, vessel density, changes in blood flow, and neovascularization. Furthermore, neurovascular coupling can be examined through OCTA assessment of retinal hemodynamics, and specific OCTA parameters have been linked to measures of visual function (e.g., BCVA). Therefore, OCTA presents a large and promising toolkit for clinical trials of patients with DR and DR-related complications. Indeed, several trials have already begun to incorporate OCTA metrics.

Despite its promise, it is clear that several challenges remain in the use of OCTA. As a relatively new technology, there is still a lack of standardized tools and procedures to address common imaging problems like artifacts, segmentation errors, and image focus. Although the use of specific scan types and patterns (including whole-vascular-layer OCT or PR-OCTA) and techniques (deep-learning algorithms and image reconstruction (Hormel et al., 2021b)) can ameliorate these problems, without a consensus on the best methodology, it can be difficult to compare results between studies, centers, and machines. However, since its advent, OCTA technology has been steadily improving; the emergence of higher-speed OCT platforms with a wider field of view is enabling more precise assessment of the peripheral retina for neovascularization and non-perfusion. Improved software that accurately delineates non-perfusion in the retinal plexuses without being affected by artifacts will make the assessment of ischemia more reliable. Assessment of macular ischemia remains a particular challenge because the FAZ size varies substantially even among the normal population. The development of algorithms that can accurately estimate the baseline FAZ area and loss of perfusion relative to that baseline will be essential to the clinically meaningful assessment of macular ischemia.

Here, we propose the following methodology for the effective use of OCTA in clinical trials of DR. Using a full-retinal slab OCTA image can limit segmentation errors and confounding factors, such as center-involved DME. However, plexus-specific detection of avascular area (a.k.a. Non-perfusion area) in the macula is more sensitive than full-retinal slab detection of avascular area. Therefore, choice of OCTA slab thickness for patients with DR should be guided by a combination of both comorbidity extent and metric of interest. In addition, given emerging data suggesting the importance of the peripheral retinal vasculature in assessing and predicting DR progression, wide-field OCTA imaging should be used. As intra-individual repeatability is quite high when using the same device type, but there is generally poor agreement for OCTA metrics between different device models (Li et al., 2018), the same OCTA device should be used to measure a patient over time, and, where possible and until better conversion metrics can be developed, across centers for clinical trials. Clinical trial protocols should account for the relatively high degree of poor-quality data likely to result from sub-optimal imaging conditions and incorporate time and processes for assessing OCTA image quality, re-training sites with an active dialogue between reading centers and sites, and re-imaging patients where absolutely necessary. Finally, the use of automated methods and algorithms for OCTA image analysis, such as those that can reconstruct images, distinguish between areas of true and false signals, and produce quantitative metrics, such as FAZ area, will greatly improve the efficiency and standardization of results between studies.

A number of informative OCTA metrics could be used to assess DR in future clinical trials. Assessing multiple measurements of the FAZ (area, acircularity, 3D para-FAZ vessel density) would provide an informative mix of quantitative and qualitative information that remains robust across longitudinal studies. In addition, while the precise relationship between vessel density, DR severity, and DR onset is under debate, there is a reasonable volume of evidence supporting a link between vessel density and DR, and vessel density may therefore be included in clinical trials of DR. Finally, with the advent of wide-field OCTA, the examination of extrafoveal avascular zones may become even more informative when assessing patients with early-stage DR. However, wide-field images remain limited by the currently available fields of view, leading to reduced density of data which can degrade measurement accuracy. Therefore, collecting higher-density, higher-resolution OCTA scans of the macula in combination with wide-field OCTA images of extrafoveal regions may provide the best sensitivity to early changes. Collecting these metrics as standard across trials would help to generate a large OCTA database of DR images, ultimately facilitating the quick and noninvasive evaluation of patients with diabetes at risk of developing DR.

Use of a standardized image format across OCTA devices is critical to establish the large OCTA datasets necessary to answer outstanding questions on OCTA metrics in DR. At the time of writing, a collaborative ocular imaging standardization initiative by the NEI, FDA, and ONC is underway (NEI-FDA-ONC, 2022). Although the outputs of this workshop are yet to be published, this ongoing initiative should assist in establishing a common foundation of imaging standards that can be applied across trials. Furthermore, the American Academy of Ophthalmology has recommended that imaging device manufacturers adhere to a set of standard formats (Lee et al., 2021). Adherence to these recommendations will facilitate collaborative research, improve data quality, and ultimately improve clinical care for patients.

When we have solved these issues and established a common set of OCTA procedures for use in clinical trials, it will be possible to realize the full potential of OCTA as a robust and regulator-accepted clinical trial endpoint.

## Figures and Tables

**Table 1:** A summary of optical coherence tomography angiography metrics used in selected previous and ongoing clinical trials for diabetic retinopathy, diabetic macular edema, or diabetic macular ischemia, including primary endpoint and measurement. Please note that this is not an exhaustive list.

Trial	Primary endpoint	Indication	Measurement	Trial status

**CORISMAP** NCT02876744	Macular and peripheral ischemia	DR	Correlation of macular and peripheral ischemia using OCTA	Completed (Jan 2021)
**ICOD** NCT04038125	Vessel perfusion	DME	Capillary reperfusion over 6 months by OCTA	Unknown
**HORNBILL** NCT04424290(Chong et al., 2022)	FAZ size	DMI	Change from baseline of the size of the FAZ using OCTA in superficial and combined vascular complex	Completed (April 2023)
NCT03922932	Non-perfusion areas (extrafoveal avascular area)	DR	PR-OCTA measure of non-perfusion (mm^2^) over 3 years Non-PR-OCTA measure of retinal non-perfusion (mm^2^) over 1 year Non-PR-OCTA of retinal neovascularization (mm^2^) over 1 year	Recruiting
NCT04660006	Vessel density/perfusion	DR/DME	Change from baseline in retinal vessel density at 3 months by OCTA Change from baseline in retinal perfusion density at 3 months by OCTA	Unknown
	FAZ size/circularity		Change from baseline in FAZ size at 3 months by OCTA	
NCT03765112	Perfusion density	DR	Perfusion density by OCTA	Recruiting
	FAZ size		FAZ size at 6 months by OCTA	
**PROPER**	Vascular density	DR	Change in vascular density of retinal capillary plexuses at 3, 6, 9, and 12 months by OCTA	RActive, not recruiting
NCT04674254	FAZ size		Change in FAZ size at 3, 6, 9, and 12 months by OCTA	
PARTRIDGE NCT04919499	FAZ size	DMI	Change from baseline in FAZ size using OCTA at 12, 16, and 20 weeks	Active, not recruiting

Trial statuses are accurate as of 8 August 2023. DME, diabetic macular edema; DMI, diabetic macular ischemia; DR, diabetic retinopathy; FAZ, foveal avascular zone; OCTA, optical coherence tomography angiography; PR-OCTA, projection-resolved optical coherence tomography angiography.

## Data Availability

No data was used for the research described in the article.

## Abbreviations

BCVA: best-corrected visual acuity
DCP: deep capillary plexus
DME: diabetic macular edema
DMI: diabetic macular ischemia
DR: diabetic retinopathy
DRIL: disorganization of the retinal inner layers
DVC: deep vascular complex
ETDRS: Early Treatment Diabetic Retinopathy Study
FAZ: foveal avascular zone
FDA: US Food and Drug Administration
ICP: intermediate capillary plexus
IRMA: intraretinal microvascular abnormality
NEI: National Eye Institute
NPDR: non-proliferative diabetic retinopathy
NVD: neovascularization of the disc
NVE: neovascularization elsewhere in the retina
OCT: optical coherence tomography
OCTA: optical coherence tomography angiography
ONC: Office of National Coordinator for Health Information Technology
PDR: proliferative diabetic retinopathy
PR: projection-resolved
SCP: superficial capillary plexus
SVP: superior vascular plexus
tbFAZ: theoretical baseline foveal avascular zone
VEGF: vascular endothelial growth factor
VISTA: variable interscan time analysis
